# Supplementary material for: Genetic analysis of water loss of excised leaves associated with drought tolerance in wheat
Source: PeerJ. 2018 Jul 6;6:e5063. doi: 10.7717/peerj.5063 (PMC6037134; doi:10.7717/peerj.5063)
Supplement: Supplemental Information 1 [file peerj-06-5063-s001.pdf]

Figure S1

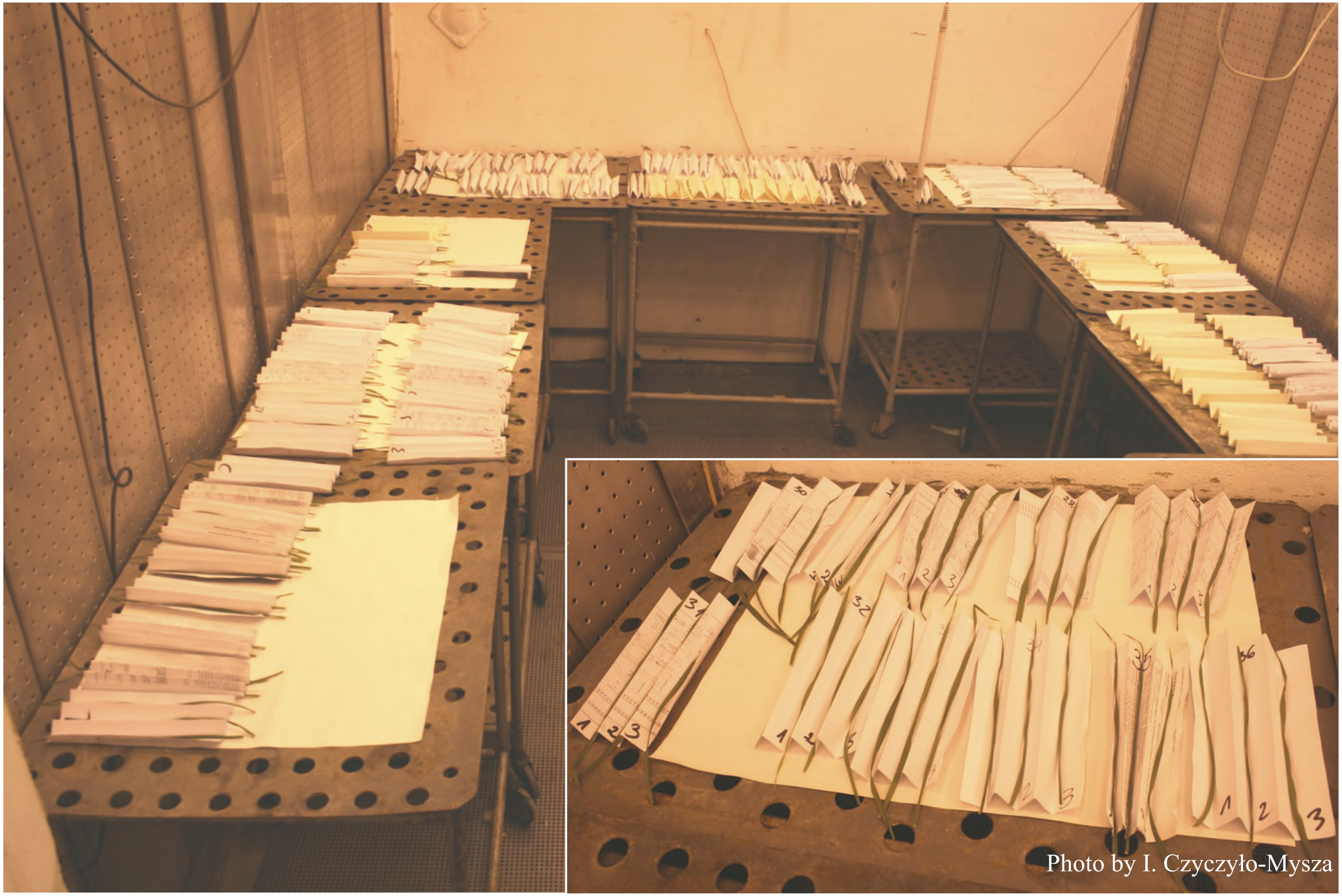

Figure S2

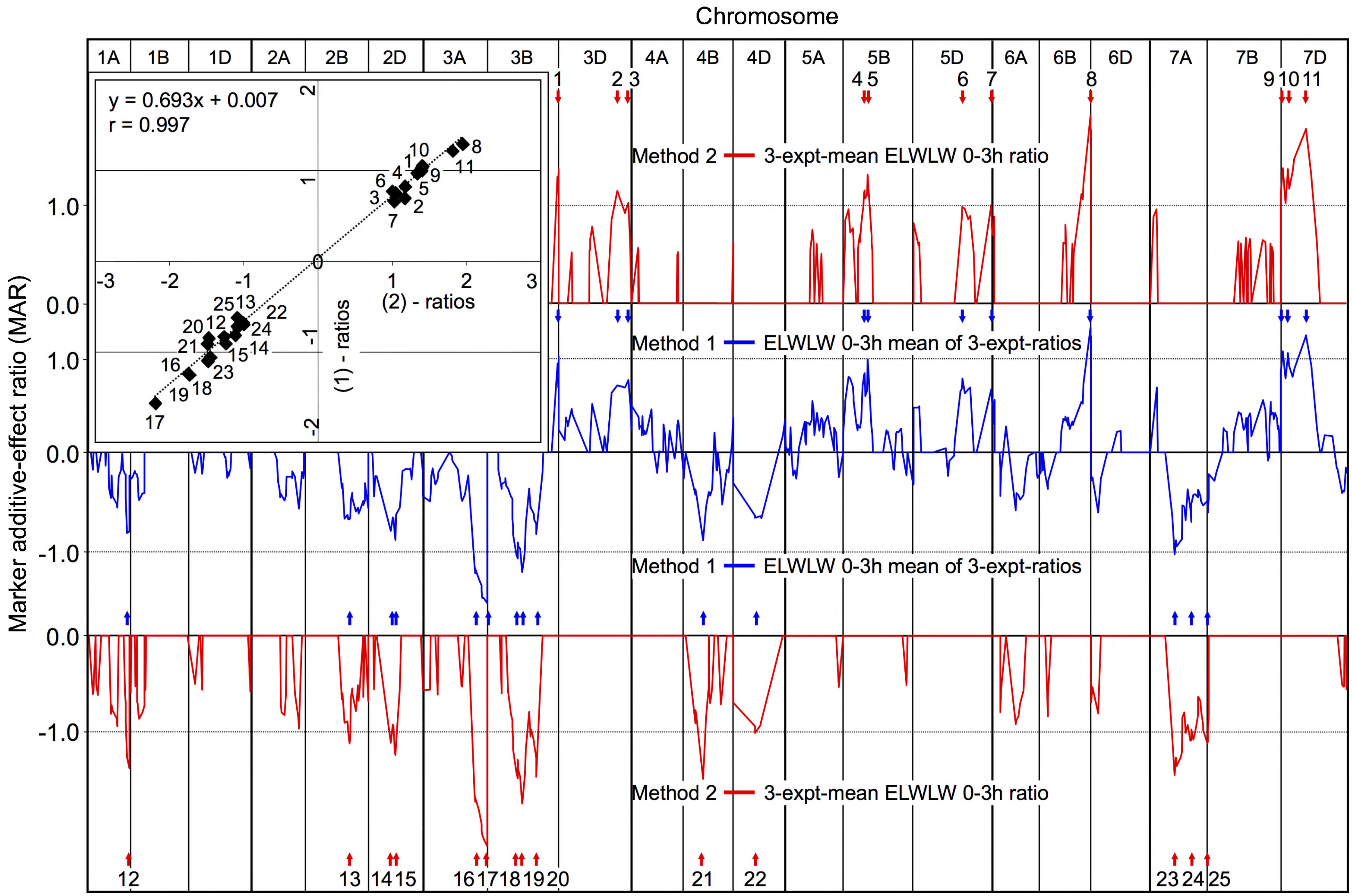
Chromosome
1A 1B 1D 2A 2B 2D 3A 3B 3D 4A 4B 4D 5A 5B 5D 6A 6B 6D 7A 7B 7D
Marker additive-effect ratio (MAR)
Method 2 3-expt-mean ELWLW 0-3h ratio
Method 1 ELWLW 0-3h mean of 3-expt-ratios
Method 1 ELWLW 0-3h mean of 3-expt-ratios
Method 2 3-expt-mean ELWLW 0-3h ratio
1.0
0.0
1.0
0.0
-1.0
0.0
-1.0
y = 0.693x + 0.007
r = 0.997
(2) - ratios
(1) - ratios
-3 -2 -1 0 1 2 3
2 1 -1 -2
1 2 3 4 5 6 7 8 9 10 11
12 13 14 15 16 17 18 19 20 21 22 23 24 25

Figure S3

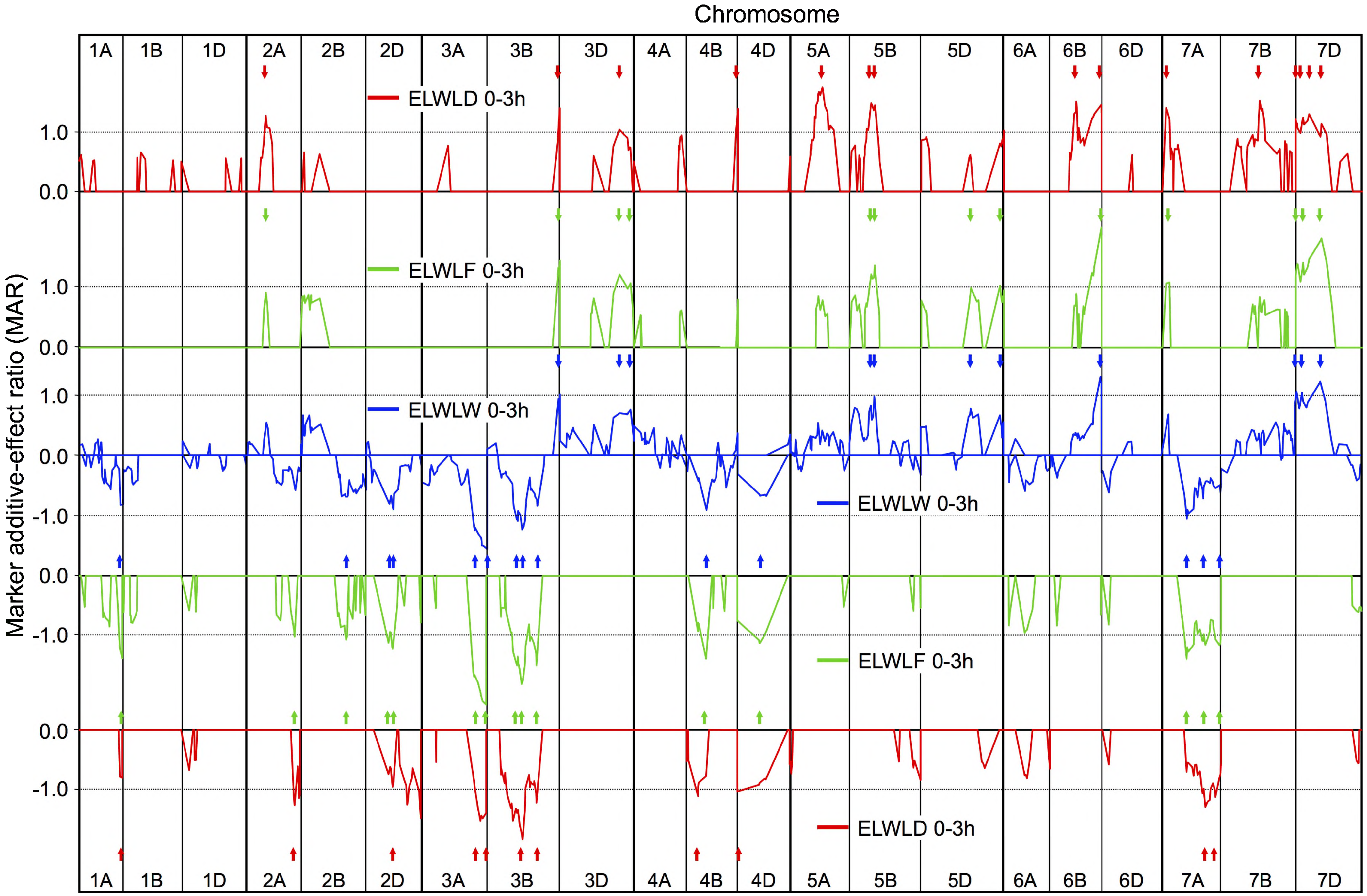
Chromosome
1A
1B
1D
2A
2B
2D
3A
3B
3D
4A
4B
4D
5A
5B
5D
6A
6B
6D
7A
7B
7D
ELWLD 0-3h
ELWLF 0-3h
ELWLW 0-3h
ELWLW 0-3h
ELWLF 0-3h
ELWLD 0-3h
Marker additive-effect ratio (MAR)
1.0
0.0
1.0
0.0
1.0
0.0
-1.0
0.0
-1.0
0.0
-1.0

Figure S4

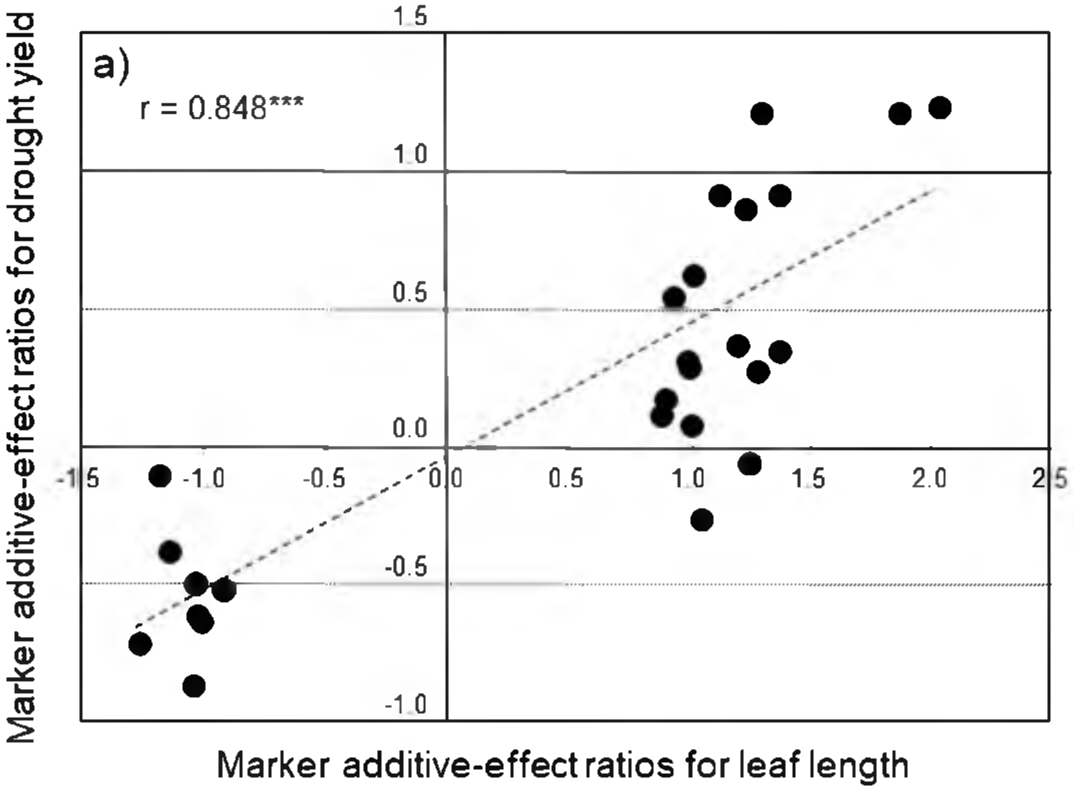

a)
r = 0.848***
Marker additive-effect ratios for drought yield
Marker additive-effect ratios for leaf length
1.5
1.0
0.5
0.0
-0.5
-1.0
-1.5
-1.0
-0.5
0.0
0.5
1.0
1.5
2.0
2.5


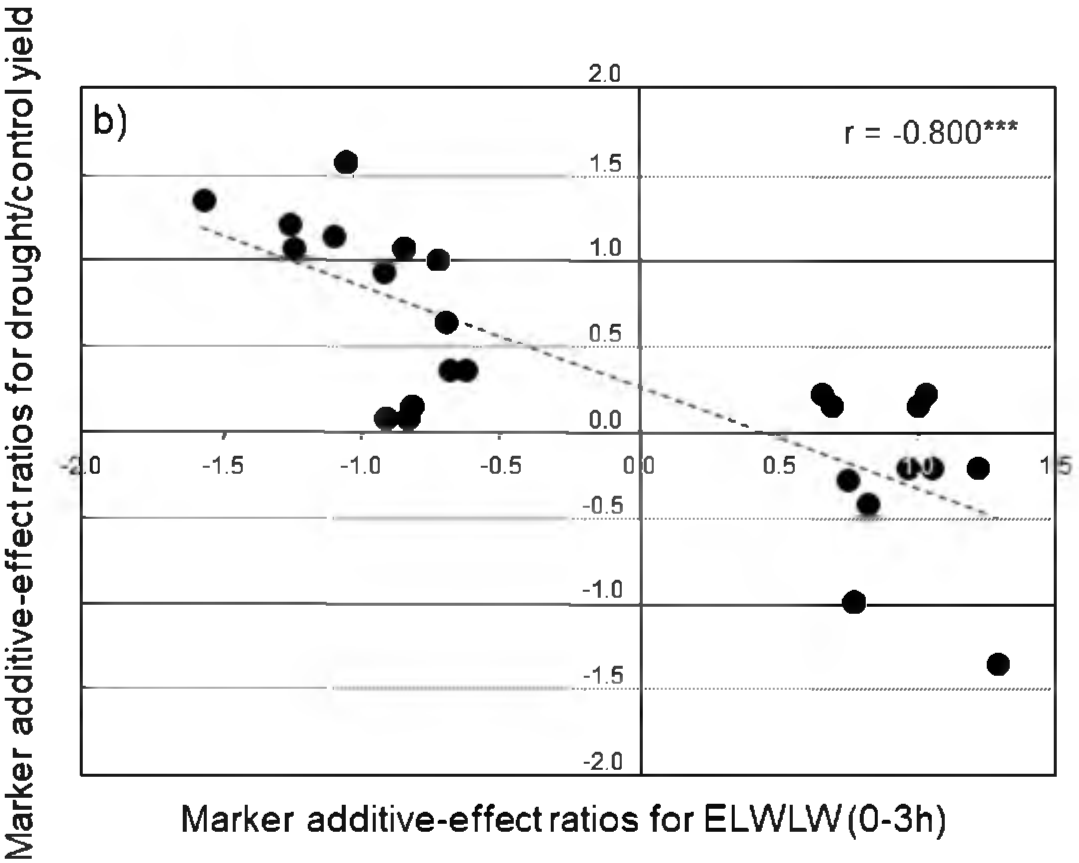

b)
r = -0.800***
Marker additive-effect ratios for drought/control yield
Marker additive-effect ratios for ELWLW (0-3h)
2.0
1.5
1.0
0.5
0.0
-0.5
-1.0
-1.5
-2.0
-2.0
-1.5
-1.0
-0.5
0.0
0.5
1.0
1.5

Figure S5

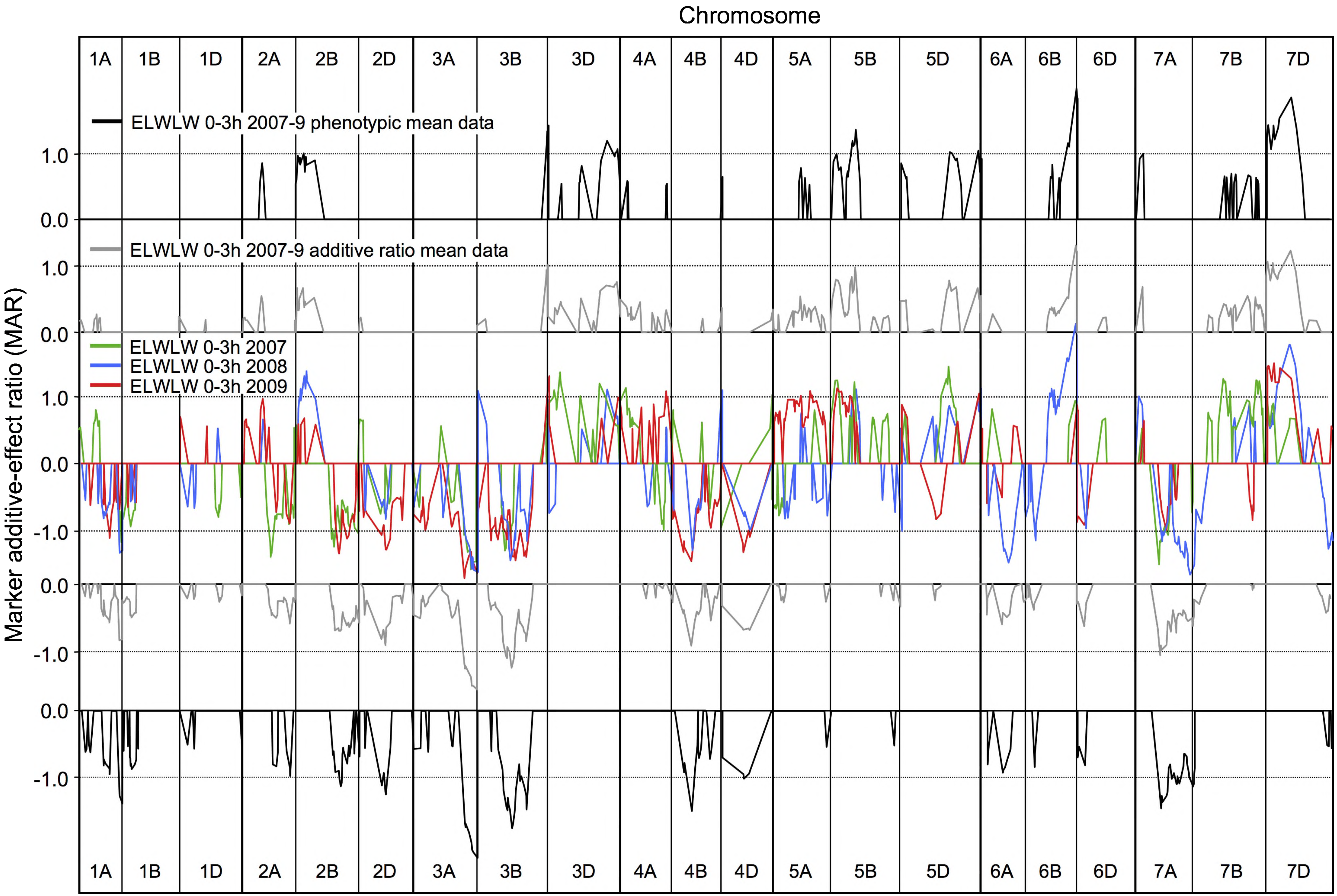
Chromosome
1A
1B
1D
2A
2B
2D
3A
3B
3D
4A
4B
4D
5A
5B
5D
6A
6B
6D
7A
7B
7D
ELWLW 0-3h 2007-9 phenotypic mean data
ELWLW 0-3h 2007-9 additive ratio mean data
ELWLW 0-3h 2007
ELWLW 0-3h 2008
ELWLW 0-3h 2009
Marker additive-effect ratio (MAR)
1.0
0.0
-1.0

Figure S6

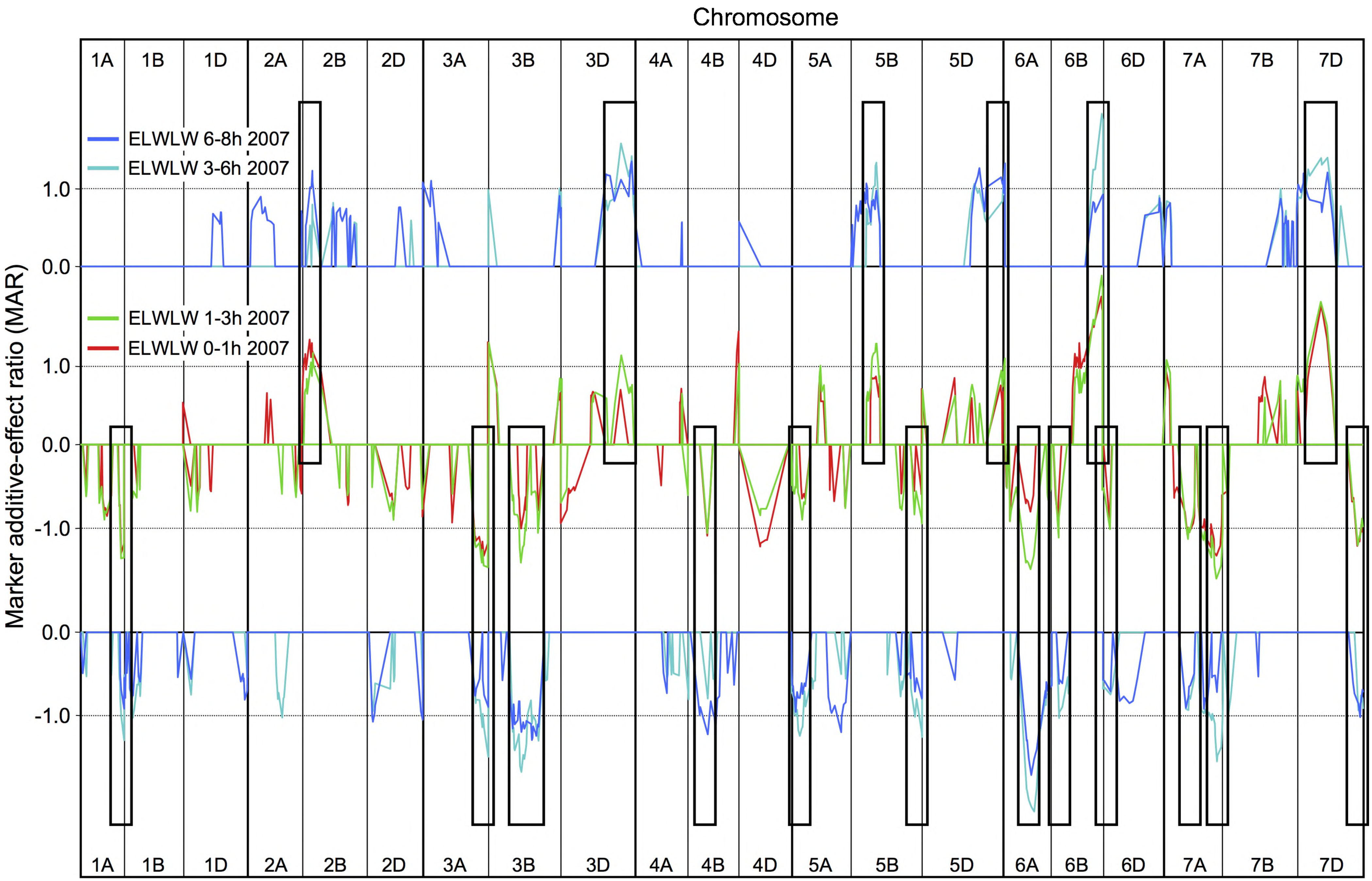

Chromosome
1A
1B
1D
2A
2B
2D
3A
3B
3D
4A
4B
4D
5A
5B
5D
6A
6B
6D
7A
7B
7D
ELWLW 6-8h 2007
ELWLW 3-6h 2007
ELWLW 1-3h 2007
ELWLW 0-1h 2007
1.0
0.0
-1.0
Marker additive-effect ratio (MAR)

**Supplementary figure legends:**

Figure S1. Excised leaf 4 of wheat plants placed in card holders in a controlled-environment cabinet. The insert shows the arrangement in more detail with three replicate leaves per CSDH line in adjacent card holders.

Figure S2. Single marker analysis (SMA) of marker additive-effect ratios (MARs) for ELWLW$_{0\text{-}3h}$ to demonstrate how QTL peak additive-effect ratios for 3-experiment ratio means were identified.

[Method 1] ELWLW 0-3h 3-expt ratio mean was derived as:

trait mean$_{I}$ →□LRmapqtl$_{I}$ → marker additive effect$_{I}$ → additive effect ratio$_{I}$ →□I-III□mean additive effect ratio.

[Method 2] 3-expt mean ELWLW 0-3h ratio was derived as:

trait mean$_{I\text{-}III}$ → (I+II+III)/3 → LRmapqtl → marker additive effect → additive effect ratio.

Continuous coloured lines join MARs for adjacent markers. Markers are ordered sequentially left to right from chromosome 1A short arm to chromosome 7D long arm. Positive MARs indicate alleles with increasing effects from Chinese Spring. Negative MARs indicate alleles with increasing effects from SQ1. Numbered arrows, coloured according to method used to calculate MARs, identify QTL peaks ≥1.0 coincident between Method 1 and Method 2. No positive MAR peaks reaching the threshold of 1.0 by either Method were present on chromosomes 1A to 3B behind the insert. Graph insert shows the relationship between additive-effect ratios calculated according to Method 2 (x-axis) and Method 1 (y-axis) at each of 25 numbered peak MARs ≥1.0 (significant at $P \leq 0.05$). The regression equation shows that significance using Method 2 was equivalent to peaks of ≥0.70 using Method 1. Peaks ≥1.0 using Method 2 and separated by at least 10 cM were considered for QTLs described in Table S1.

Figure S3. Single marker analysis (SMA) of marker additive-effect ratios (MARs) for ELWLW$_{0\text{-}3h}$, ELWLF$_{0\text{-}3h}$ and ELWLD$_{0\text{-}3h}$. Arrows indicate MAR peaks ≥1.0 coincident with ELWL$_{0\text{-}3h}$ calculated on another basis. Other details as for Figure S2.

Figure S4a, b. Associations between peak marker additive-effect ratios (MARs) significant at $P \leq 0.05$ for a) leaf length (26 significant peak MARs) and 12-trial mean drought yield, and b) $ELWLW_{0\text{-}3h}$ (25 significant peak MARs) and drought/control yield ratio. For each abscissa (leaf length and $ELWLW_{0\text{-}3h}$), the nearest peak MARs for the corresponding ordinate (drought yield and drought/control yield ratio) within 10 cM of the abscissa significant peak MARs was used to calculate linear regressions.

Figure S5. Single marker analysis (SMA) of marker additive-effect ratios (MARs) for $ELWLW_{0\text{-}3h}$ in each year (2007, 2008, 2009) compared with mean $ELWLW_{0\text{-}3h}$ calculated using Method 1 (ELWLW 0-3h 2007-9 additive ratio mean data) and Method 2 (ELWLW 0-3h 2007-9 phenotypic mean data). Other details as for Figure S2.

Figure S6. Single marker analysis (SMA) of marker additive-effect ratios (MARs) for ELWLW in 2007 for time intervals 0-1 h, 1-3 h, 3-6 h and 6-8 h. Areas of coincidence of major peak MARs (at least *ca.* ±0.7) for all four time intervals are boxed. To aid clarity, only MARs ≥0.5 are shown. Other details as for Figure S2.
