# Supplementary material for: Genetic analysis of water loss of excised leaves associated with drought tolerance in wheat
Source: PeerJ. 2018 Jul 6;6:e5063. doi: 10.7717/peerj.5063 (PMC6037134; doi:10.7717/peerj.5063)
Supplement: Table S1 — SMA 3-year (experiment)-mean ratios for traits are the 3-experiment-means of experiment additive effect ratios calculated as marker additive effect/minimum absolute marker additive effect significant at P < 0.05. Three-year (experiment)-mean SMA ratios are calculated as above from a single LRmapqtl analysis of 3-experiment-mean data for each trait, for which the level of significance is provided by LRmapqtl. QTLs for SMA 3-year-mean ratios less than 1.0 are shown in italics. QTLs in bold were present also using CIM. I, II and III indicate results for 2007, 2008, 2009 respectively.§Numbers in parentheses below trait names are minimum absolute additive effects for P < 0.05 significance with LRmapqtl. The upper value refers to the mean of minimum significant additive effects for each experiment [Method 1]. The lower value refers to the minimum significant additive effect using the 3-year mean phenotypic data for a single analysis with LRmapqtl [Method 2] (only experiment III, 2009 for midrib thickness). [file peerj-06-5063-s002.doc]

Table S1. Characteristics of QTLs identified using SMA with LRmapqtl for four ELWL and four leaf traits. SMA 3-year (experiment)-mean ratios for traits are the 3-experiment-means of experiment additive effect ratios calculated as marker additive effect/minimum absolute marker additive effect significant at *P*<0.05. Three-year (experiment)-mean SMA ratios are calculated as above from a single LRmapqtl analysis of 3-experiment-mean data for each trait, for which the level of significance is provided by LRmapqtl. QTLs for SMA 3-year-mean ratios less than 1.0 are shown in italics. QTLs in bold were present also using CIM. I, II and III indicate results for 2007, 2008, 2009 respectively.

| **Trait and minimum significant additive effects**^§^ | QTL designation | Peak marker | **cM from first marker** | **Chromosome + bin location** | **SMA 3-year- mean ratio** | **Years QTL ≥1.0 present** | **Additive effect 3-year-mean** | **3-year-mean SMA ratio** | **3-year-mean additive effect** |
| --- | --- | --- | --- | --- | --- | --- | --- | --- | --- |
| **ELWLW**_0-3_  (2.031)^§^  (1.466) | *QELWLW_0-3_.csdh-1A* | *wPt-2976* | *115.7* | *1AL 0.61-1.00* | *-0.836* | I, II | -1.698 | *-1.377*** | -2.019 |
|  | *QELWLW_0-3_.csdh-2B* | *psr1870* | *127.3* | *2BL 0.36-0.50* | *-0.696* | *II, III* | *-1.413* | *-1.121** | *-1.643* |
|  | *QELWLW_0-3_.csdh-2D.1* | *wmc453.4* | *65.0* | *2DS 0.33-0.47* | *-0.818* | *III* | *-1.661* | *-1.111** | *-1.629* |
|  | *QELWLW_0-3_.csdh-2D.2* | *m72p78.4a* | *76.8* | *2DS ~0.33* | *-0.909* | *III* | *-1.847* | *-1.241** | *-1.819* |
|  | *QELWLW_0-3_.csdh-3A.1* | gwm480 | 150.9 | 3AL ~0.78 | -1.255 | I, II, III | -2.548 | -1.728*** | -2.533 |
|  | ***QELWLW_0-3_.csdh-3A.2*** | **wPt-733571** | **186.1** | **3AL 0.78-1.00** | **-1.567** | **I, II, III** | **-3.183** | **-2.188****** | **-3.208** |
|  | *QELWLW_0-3_.csdh-3B.1* | wmc231 | 87.1 | 3BS 0.57-0.78 | -1.102 | II, III | -2.237 | -1.485** | -2.177 |
|  | *QELWLW_0-3_.csdh-3B.2* | wPt-4048 | 99.9 | 3BL C-0.22 | -1.244 | I, II, III | -2.526 | -1.744*** | -2.557 |
|  | *QELWLW_0-3_.csdh-3B.3* | *wPt-4222* | *142.5* | *3BL 0.63-1.00* | *-0.849* | *I, III* | *-1.723* | *-1.466*** | *-2.149* |
|  | *QELWLW_0-3_.csdh-3B.4* | m17p65.2 | 207.2 | 3BL 0.63-1.00 | 1.002 | III | 2.035 | 1.398** | 2.049 |
|  | *QELWLW_0-3_.csdh-3D.1* | *m83p65.9* | *169.6* | *3DL 0.51-0.81* | *0.695* | *I* | *1.412* | *1.171** | *1.717* |
|  | *QELWLW_0-3_.csdh-3D.2* | *wPt-730886* | *200.0* | *3DL 0.81-1.00* | *0.752* | *-* | *1.526* | *1.048** | *1.536* |
|  | *QELWLW_0-3_.csdh-4B* | *psp3163* | *54.9* | *4BS 0.37-0.57* | *-0.916* | *I, III* | *-1.860* | *-1.488*** | *-2.181* |
|  | *QELWLW_0-3_.csdh-4D* | *wPt-2379* | *63.0* | *4D-C* | *-0.678* | *III* | *-1.378* | *-1.009** | *-1.479* |
|  | *QELWLW_0-3_.csdh-5B.1* | *psr725* | *61.3* | *5BL 0.55-0.75* | *0.823* | *-* | *1.672* | *1.173** | *1.720* |
|  | *QELWLW_0-3_.csdh-5B.2* | *psp3037* | *70.7* | *5BL 0.55-0.75* | *0.966* | *I, II* | *1.963* | *1.337*** | *1.960* |
|  | *QELWLW_0-3_.csdh-5D.1* | *cfd3* | *141.5* | *5DL 0.60-0.74* | *0.768* | *II* | *1.561* | *1.003** | *1.470* |
|  | ***QELWLW_0-3_.csdh-5D.2*** | ***m92p78.9*** | ***224.5*** | ***5DL 0.76-1.00*** | ***0.658*** | ***III*** | ***1.336*** | ***1.024**** | ***1.501*** |
|  | ***QELWLW_0-3_.csdh-6B*** | **wPt-4164** | **143.0** | **6BL 0.40-1.00** | **1.295** | **I** | **2.629** | **1.948****** | **2.856** |
|  | ***QELWLW_0-3_.csdh-7A.1*** | **wPt-744074** | **72.2** | **7AS 0.59-0.89** | **-1.060** | **I, II** | **-2.152** | **-1.450**** | **-2.126** |
|  | *QELWLW_0-3_.csdh-7A.2* | *barc108.1* | *118.9* | *7AL C-0.39* | *-0.723* | *I* | *-1.469* | *-1.085** | *-1.591* |
|  | *QELWLW_0-3_.csdh-7A.3* | *psp3094.1* | *167.9* | *7AL 0.86-0.90* | *-0.624* | *I* | *-1.268* | *-1.086** | *-1.592* |
|  | ***QELWLW_0-3_.csdh-7D.1*** | **gwm635.1** | **4.4** | **7DS 0.61-1.00** | **1.051** | **III** | **2.134** | **1.408**** | **2.064** |
|  | *QELWLW_0-3_.csdh-7D.2* | wPt-743501 | 19.8 | 7DS 0.61-1.00 | 1.033 | III | 2.099 | 1.399** | 2.051 |
|  | *QELWLW_0-3_.csdh-7D.3* | mgl59 | 71.8 | 7DS 0.36-0.61 | 1.217 | I, III | 2.472 | 1.817*** | 2.663 |
| **ELWLA**_0-3_ (0.133)^§^  (0.095) | *QELWLA_0-3_.csdh-1A.1* | *wPt-665693* | *87.0* | *1AL 0.61-1.00* | *-0.721* | *II* | *-0.096* | *-1.021** | *-0.097* |
|  | *QELWLA_0-3_.csdh-1A.2* | m69p78.4 | 123.6 | *1AL 0.61-1.00* | -1.050 | I, II | -0.140 | -1.579** | -0.150 |
|  | *QELWLA_0-3_.csdh-1D* | *wmc432* | *22.3* | *1DS 0.70-1.00* | *-0.769* | *I, II* | *-0.102* | *-1.263** | *-0.120* |
|  | *QELWLA_0-3_.csdh-2A.1* | *wPt-5029* | *57.6* | *2AS C-0.78* | *0.793* | *II* | *0.105* | *1.105** | *0.105* |
|  | *QELWLA_0-3_.csdh-2A.2* | psp2151.3 | 139.3 | 2AL 0.85-1.00 | -1.070 | I, III | -0.142 | -1.463** | -0.139 |
|  | *QELWLA_0-3_.csdh-2B.1* | *wPt-0335* | *108.1* | *2B-C* | *-0.813* | *-* | *-0.108* | *-1.147** | *-0.109* |
|  | ***QELWLA_0-3_.csdh-2B.2*** | ***psr1870*** | ***127.3*** | ***2BL 0.36-0.50*** | ***-0.718*** | ***III*** | ***-0.095*** | ***-1.126**** | ***-0.107*** |
|  | *QELWLA_0-3_.csdh-2D* | *wPt-665644* | *78.9* | *2DS C-0.33* | *-0.721* | *I, III* | *-0.096* | *-1.095** | *-0.104* |
|  | *QELWLA_0-3_.csdh-3A.1* | wPt-4398 | 161.8 | 3AL 0.78-1.00 | -1.097 | I, III | -0.146 | -1.537** | -0.146 |
|  | *QELWLA_0-3_.csdh-3A.2* | wPt-2813 | 174.5 | 3AL 0.78-1.00 | -1.076 | I, III | -0.143 | -1.516** | -0.144 |
|  | *QELWLA_0-3_.csdh-3B.1* | *wPt-6239* | *75.4* | *3BS 0.57-0.78* | *-0.959* | *II* | *-0.128* | *-1.337*** | *-0.127* |
|  | *QELWLA_0-3_.csdh-3B.2* | *wmc1* | *103.1* | *3BL C-0.22* | *-0.887* | *-* | *-0.118* | *-1.242** | *-0.118* |
|  | *QELWLA_0-3_.csdh-3B.3* | *m17p65.2* | *207.2* | *3BL 0.63-1.00* | *0.742* | *-* | *0.099* | *1.011** | *0.096* |
|  | *QELWLA_0-3_.csdh-3D* | *m49p78.5* | *151.5* | *3DL 0.51-0.81* | *0.753* | *-* | *0.100* | *1.042** | *0.099* |
|  | ***QELWLA_0-3_.csdh-4B.1*** | ***wPt-1400*** | ***31.8*** | ***4BS 0.81-1.00*** | ***-0.625*** | ***III*** | ***-0.083*** | ***-1.021**** | ***-0.097*** |
|  | *QELWLA_0-3_.csdh-4B.2* | *dupw043* | *143.3* | *4BL 0.86-1.00* | *0.625* | *I* | *0.083* | *1.095** | *0.104* |
|  | *QELWLA_0-3_.csdh-4D* | *gwm609* | *149.2* | *4DL 0.71-1.00* | *0.882* | *II* | *0.117* | *1.263** | *0.120* |
|  | *QELWLA_0-3_.csdh-5A.1* | *psr120.1* | *70.0* | *5AL 0.57-0.78* | *0.677* | *III* | *0.090* | *1.095** | *0.104* |
|  | ***QELWLA_0-3_.csdh-5A.2*** | **vrn-A1** | **91.1** | **5AL 0.58-0.78** | **1.253** | **I, III** | **0.167** | **1.737***** | **0.165** |
|  | *QELWLA_0-3_.csdh-5B.1* | *wPt-1261* | *27.1* | *5BS 0.81-1.00* | *0.890* | *II, III* | *0.118* | *1.221** | *0.116* |
|  | ***QELWLA_0-3_.csdh-5B.2*** | **wPt-7418** | **60.2** | **5BL 0.55-0.75** | **1.225** | **I, II** | **0.163** | **1.811***** | **0.172** |
|  | *QELWLA_0-3_.csdh-6B.1* | *barc101.2* | *72.0* | *6B-C* | *0.932* | *I* | *0.124* | *1.305*** | *0.124* |
|  | *QELWLA_0-3_.csdh-6B.2* | *m43p78.13* | *92.0* | *6BL 0.40-1.00* | *0.865* | *I* | *0.115* | *1.232** | *0.117* |
|  | ***QELWLA_0-3_.csdh-6B.3*** | ***wPt-4164*** | ***143.0*** | ***6BL 0.40-1.00*** | ***0.946*** | ***I, II*** | ***0.126*** | ***1.589***** | ***0.151*** |
|  | *QELWLA_0-3_.csdh-6D* | *barc173* | *21.8* | *6DS 0.99-1.00* | *-0.718* | *I* | *-0.095* | *-1.074** | *-0.102* |
|  | *QELWLA_0-3_.csdh-7A* | *m39p78.4* | *20.2* | *7AS 0.89-1.00* | *0.911* | *I, III* | *0.121* | *1.253** | *0.119* |
|  | *QELWLA_0-3_.csdh-7B* | wPt-5070 | 107.3 | 7BL 0.78-1.00 | 1.014 | II | 0.135 | 1.442** | 0.137 |
|  | *QELWLA_0-3_.csdh-7D.1* | wPt-2926 | 2.2 | 7DS 0.61-1.00 | 1.123 | I, II, III | 0.149 | 1.568** | 0.149 |
|  | ***QELWLA_0-3_.csdh-7D.2*** | **wPt-671748** | **23.3** | **7DS 0.61-1.00** | **1.157** | **I, II, III** | **0.154** | **1.621**** | **0.154** |
|  | *QELWLA_0-3_.csdh-7D.3* | *mgl59* | *71.8* | *7DS 0.36-0.61* | *0.949* | *I* | *0.126* | *1.347*** | *0.128* |
| **ELWLW**_3-6_  (3.970)^§^  (3.237) | *QELWLW_3-6_.csdh-1A* | m69p78.4 | 123.6 | 1AL 0.61-1.00 | -1.142 | I, II | -4.534 | -1.391** | -4.502 |
|  | *QELWLW_3-6_.csdh-1B* | m87p78.8 | 25.1 | 1B 1.06-sat 1.00 | -1.022 | I, II | -4.059 | -1.235* | -3.998 |
|  | ***QELWLW_3-6_.csdh-2A*** | **wPt-665330** | **99.4** | **2AL C-0.85** | **-1.133** | **I, II, III** | **-4.499** | **-1.384**** | **-4.480** |
|  | *QELWLW_3-6_.csdh-3A.1* | gwm480 | 150.9 | 3AL ~0.78 | -1.304 | I, II, III | -5.178 | -1.587** | -5.136 |
|  | *QELWLW_3-6_.csdh-3A.2* | wPt-733571 | 186.1 | 3AL 0.78-1.00 | -1.647 | I, II, III | -6.537 | -2.018*** | -6.533 |
|  | *QELWLW_3-6_.csdh-3B.1* | wPt-0371 | 83.9 | 3BS 0.57-0.78 | -1.445 | I, II, III | -5.737 | -1.781*** | -5.765 |
|  | ***QELWLW_3-6_.csdh-3B.2*** | **wmc1** | **103.1** | **3BL C-0.22** | **-1.712** | **I, II, III** | **-6.798** | **-2.114****** | **-6.844** |
|  | *QELWLW_3-6_.csdh-3B.3* | wPt-4222 | 142.5 | 3BL 0.63-1.00 | -1.323 | I, III | -5.252 | -1.652*** | -5.348 |
|  | *QELWLW_3-6_.csdh-3B.4* | *wPt-7614* | *202.7* | *3BL 0.63-1.00* | *0.816* | *I* | *3.238* | *1.005** | *3.240* |
|  | *QELWLW_3-6_.csdh-3D* | wPt-730886 | 200.0 | 3DL 0.81-1.00 | 1.219 | I, III | 4.838 | 1.525** | 4.937 |
|  | *QELWLW_3-6_.csdh-4B* | psp3163 | 54.9 | 4BS 0.37-0.57 | -1.113 | III | -4.417 | -1.376** | -4.454 |
|  | *QELWLW_3-6_.csdh-5A* | *m43p78.10* | *19.6* | *5AL ~0.35* | *-0.784* | *I, II* | *-3.111* | *-1.001** | *-3.101* |
|  | *QELWLW_3-6_.csdh-5B.1* | *wPt-1951* | *47.4* | *5BL 0.29-0.55* | *0.929* | *III* | *3.687* | *1.137** | *3.682* |
|  | *QELWLW_3-6_.csdh-5B.2* | psr725 | 61.3 | 5BL 0.55-0.75 | 1.075 | II, III | 4.267 | 1.310* | 4.241 |
|  | *QELWLW_3-6_.csdh-5B.3* | psp3037 | 70.7 | 5BL 0.55-0.75 | 1.180 | I, II | 4.684 | 1.432** | 4.637 |
|  | *QELWLW_3-6_.csdh-5D* | *gwm292* | *158.2* | *5DL 0.76-1.00* | *0.916* | *I, III* | *3.636* | *1.151** | *3.726* |
|  | ***QELWLW_3-6_.csdh-6A*** | **wPt-0902** | **68.4** | **6AL C-0.55** | **-1.521** | **I, II, III** | **-6.038** | **-1.914***** | **-6.194** |
|  | *QELWLW_3-6_.csdh-6B* | wPt-4164 | 143.0 | 6BL 0.40-1.00 | 1.346 | I, II | 5.342 | 1.642** | 5.314 |
|  | *QELWLW_3-6_.csdh-7A.1* | *wPt-744074* | *72.2* | *7AS 0.59-0.89* | *-0.856* | *I, II* | *-3.397* | *-1.157** | *-3.745* |
|  | *QELWLW_3-6_.csdh-7A.2* | *barc108.1* | *118.9* | *7AL C-0.39* | *-0.956* | *I* | *-3.794* | *-1.171** | *-3.790* |
|  | *QELWLW_3-6_.csdh-7A.3* | *psp3094.1* | *167.9* | *7AL 0.86-0.90* | *-0.833* | *I, II* | *-3.307* | *-1.054** | *-3.413* |
|  | ***QELWLW_3-6_.csdh-7D.1*** | **gwm635.1** | **4.4** | **7DS 0.61-1.00** | **1.108** | **II, III** | **4.401** | **1.353**** | **4.380** |
|  | ***QELWLW_3-6_.csdh-7D.2*** | **wPt-744300** | **18.6** | **7DS 0.61-1.00** | **1.211** | **I, II, III** | **4.809** | **1.482**** | **4.796** |
|  | *QELWLW_3-6_.csdh-7D.3* | barc154 | 69.7 | 7DS ~0.61 | 1.181 | I, II, III | 4.690 | 1.458** | 4.720 |
| **ELWLA**_3-6_  (0.076)^§^  (0.057) | *QELWLA_3-6_.csdh-1A* | *wPt-2976* | *115.7* | *1AL 0.61-1.00* | *-0.876* | *II* | *-0.0672* | *-1.018** | *-0.058* |
|  | *QELWLA_3-6_.csdh-1B.1* | *m87p78.8* | *25.1* | 1B 1.06-sat 1.00 | *-0.651* | *II* | *-0.0499* | *-1.000** | *-0.057* |
|  | *QELWLA_3-6_.csdh-1B.2* | *psr325.2* | *95.4* | *1BL 0.47-0.69* | *0.875* | *III* | *0.0671* | *1.211** | *0.069* |
|  | *QELWLA_3-6_.csdh-1D* | *wmc432* | *22.3* | *1DS 0.70-1.00* | *-0.808* | *-* | *-0.0620* | *-1.070** | *-0.061* |
|  | *QELWLA_3-6_.csdh-2A* | wPt-665330 | 99.4 | 2AL C-0.85 | -1.033 | I, III | -0.0792 | -1.439** | -0.082 |
|  | *QELWLA_3-6_.csdh-2B.1* | *barc124.3* | *3.2* | *2BS 0.84-1.00* | *-0.845* | *I* | *-0.0648* | *-1.140** | *-0.065* |
|  | *QELWLA_3-6_.csdh-2B.2* | *wPt-7619* | *55.8* | *2BS C-0.53* | *-0.622* | *-* | *-0.0477* | *-1.035** | *-0.059* |
|  | *QELWLA_3-6_.csdh-2D* | *wPt-2761* | *97.0* | *2DL C-0.49* | *0.714* | *III* | *0.0547* | *1.088** | *0.062* |
|  | *QELWLA_3-6_.csdh-3B.1* | wPt-10948 | 74.3 | 3BS 0.57-0.78 | -1.107 | I, III | -0.0849 | -1.509** | -0.086 |
|  | *QELWLA_3-6_.csdh-3B.2* | *wmc1* | *103.1* | *3BL C-0.22* | *-0.978* | *II, III* | *-0.0750* | *-1.333*** | *-0.076* |
|  | *QELWLA_3-6_.csdh-3B.3* | *wmc326* | *167.3* | *3BL 0.63-1.00* | *-0.681* | *III* | *-0.0522* | *-1.123** | *-0.064* |
|  | *QELWLA_3-6_.csdh-3D.1* | *gwm383* | *139.2* | *3DL 0.27-0.51* | *0.938* | *I* | *0.0719* | *1.298** | *0.074* |
|  | *QELWLA_3-6_.csdh-3D.2* | *wPt-730886* | *200.0* | *3DL 0.81-1.00* | *0.777* | *I, III* | *0.0596* | *1.316*** | *0.075* |
|  | ***QELWLA_3-6_.csdh-4A*** | ***wmc262*** | ***56.7*** | ***4AL 0.80-1.00*** | ***0.582*** | ***I*** | ***0.0446*** | ***1.000**** | ***0.057*** |
|  | ***QELWLA_3-6_.csdh-5A.1*** | ***wPt-798459*** | ***2.3*** | ***5AS 0.75-0.98*** | ***-0.952*** | ***I, II*** | ***-0.0730*** | ***-1.351***** | ***-0.077*** |
|  | ***QELWLA_3-6_.csdh-5A.2*** | **barc180** | **14.3** | **5AL C-0.35** | **-1.315** | **I, II** | **-0.1008** | **-1.702***** | **-0.097** |
|  | *QELWLA_3-6_.csdh-5A.3* | *vrn-A1* | *91.1* | *5AL 0.57-0.78* | *0.769* | *II, III* | *0.0589* | *1.175** | *0.067* |
|  | ***QELWLA_3-6_.csdh-5B.1*** | **wPt-1951** | **47.4** | **5BL 0.29-0.55** | **1.192** | **I, II** | **0.0914** | **1.544**** | **0.088** |
|  | *QELWLA_3-6_.csdh-5B.2* | psr725 | 61.3 | 5BL 0.55-0.75 | 1.070 | II | 0.0820 | 1.333** | 0.076 |
|  | *QELWLA_3-6_.csdh-5B.3* | *psr806.2* | *72.8* | *5BL 0.55-0.75* | *0.929* | *II* | *0.0712* | *1.175** | *0.067* |
|  | *QELWLA_3-6_.csdh-5B.4* | *psr375.2* | *201.7* | *5BL 0.79-1.00* | *-0.878* | *III* | *-0.0673* | *-1.193** | *-0.068* |
|  | ***QELWLA_3-6_.csdh-6A*** | **wPt-0902** | **68.4** | **6AL C-0.55** | **-1.407** | **I, II, III** | **-0.1079** | **-1.930***** | **-0.110** |
|  | *QELWLA_3-6_.csdh-7A* | *wPt-5092* | *23.7* | *7AS 0.89-1.00* | *0.609* | *-* | *0.0467* | *1.000** | *0.057* |
|  | *QELWLA_3-6_.csdh-7D.1* | *wPt-2926* | *2.2* | *7DS 0.61-1.00* | *0.802* | *II* | *0.0615* | *1.018** | *0.058* |
|  | *QELWLA_3-6_.csdh-7D.2* | wPt-744300 | 18.6 | 7DS 0.61-1.00 | 1.005 | II | 0.0770 | 1.316** | 0.075 |
| **Leaf length**  (0.725) ^§^  (0.646) | *QLflength.csdh-1A.1* | wPt-733091 | 85.9 | 1AL 0.61-1.00 | 1.020 | II, III | 0.740 | 1.152*** | 0.740 |
|  | *QLflength.csdh-1A.2* | *m69p78.4* | *123.6* | *1AL 0.61-1.00* | *0.934* | II, III | 0.677 | 1.048* | 0.677 |
|  | *QLflength.csdh-1B.1* | wPt-8682 | 27.7 | 1BS 0.50-1.06 | 1.126 | I, II, III | 0.816 | 1.265* | 0.817 |
|  | *QLflength.csdh-1B.2* | wPt-1613 | 45.5 | 1BS C-0.50 | 1.295 | I, II, III | 0.939 | 1.457** | 0.914 |
|  | *QLflength.csdh-1B.3* | wPt-4532 | 143.8 | 1BL 0.85-1.00 | 1.048 | I, III | 0.760 | 1.187* | 0.767 |
|  | *QLflength.csdh-1D.1* | wPt-732602 | 108.2 | 1DL 0.41-1.00 | -1.013 | I | -0.735 | -1.135* | -0.733 |
|  | *QLflength.csdh-1D.2* | wPt-734057 | 117.2 | 1DL 0.41-1.00 | -1.032 | III | -0.748 | -1.159* | -0.733 |
|  | *QLflength.csdh-2A* | *wPt-4197* | *11.2* | *2AS 0.78-1.00* | *0.884* | III | 0.641 | 1.000* | 0.646 |
|  | *QLflength.csdh-2B.1* | wPt-7619 | 55.8 | 2BS C-0.53 | -1.270 | I, II, III | -0.921 | -1.424** | -0.920 |
|  | *QLflength.csdh-2B.2* | barc7 | 91.1 | 2BS 0.27-0.53 | -1.144 | I, II | -0.829 | -1.274* | -0.823 |
|  | ***QLflength.csdh-3A.1*** | **gwm2** | **76.6** | **3AS 0.45-1.00** | **1.456** | **I, II, III** | **1.056** | **1.642**** | **1.061** |
|  | *QLflength.csdh-3A.2* | *wPt-734079* | *113.5* | *3AL 0.42-0.85* | *0.996* | III | 0.722 | 1.130* | 0.730 |
|  | ***QLflength.csdh-3D*** | ***wPt-4569*** | ***38.9*** | ***3DS 0.55-1.00*** | ***-0.921*** | **II** | **-0.668** | **-1.028*** | **-0664** |
|  | ***QLflength.csdh-4B.1*** | **wPt-6149** | **70.9** | **4BS C-0.37** | **1.866** | **I, II, III** | **1.353** | **2.099****** | **1.356** |
|  | ***QLflength.csdh-4B.2*** | **psp3030.2** | **77.5** | **4BL C-0.71** | **2.030** | **I, II, III** | **1.472** | **2.280****** | **1.473** |
|  | *QLflength.csdh-4B.3* | wPt-3326 | 107.1 | 4BL 0.86-1.00 | 1.375 | I, II, III | 0.997 | 1.546** | 0.999 |
|  | *QLflength.csdh-4D* | *wmc473* | *78.1* | *4DL 0.31-0.56* | *0.899* | III | 0.652 | 1.014 | 0.655 |
|  | *QLflength.csdh-5A.1* | wPt-9094 | 60.4 | 5AL 0.57-0.78 | 1.195 | I, II, III | 0.866 | 1.336** | 0.863 |
|  | ***QLflength.csdh-5A.2*** | **psr426.1** | **98.5** | **5AL 0.57-0.78** | **1.370** | **I, II, III** | **0.993** | **1.536**** | **0.992** |
|  | *QLflength.csdh-5A.3* | psr145 | 111.1 | 5AL 0.57-0.78 | 1.230 | I, II, III | 0.892 | 1.381** | 0.892 |
|  | *QLflength.csdh-5D.1* | m77p64.8 | 148.3 | 5DL 0.74-0.76 | -1.037 | I, II | -0.752 | -1.156* | -0.747 |
|  | *QLflength.csdh-5D.2* | gwm212 | 162.7 | 5DL 0.76-1.00 | -1.051 | I, II | -0.762 | -1.170* | -0.756 |
|  | *QLflength.csdh-6D* | cfd80 | 75.8 | 6DL C-0.29 | -1.190 | II, III | -0.863 | -1.339** | -0.865 |
|  | *QLflength.csdh-7A* | wPt-3883 | 74.3 | 7AS 0.59-0.89 | 1.006 | I, III | 0.729 | 1.138* | 0.735 |
|  | *QLflength.csdh-7B.1* | tPt-7360 | 15.8 | 7BS 0.27-1.00 | 1.250 | I, II | 0.907 | 1.398** | 0.903 |
|  | *QLflength.csdh-7B.2* | wPt-3402 | 209.1 | 7BL 0.78-1.00 | 1.007 | II | 0.730 | 1.127* | 0.728 |
| **Leaf width**  (0.068)^§^  (0.016) | *QLfwidth.csdh-1A* | *m69p78.4* | *123.6* | 1AL 0.61-1.00 | 0.706 | II | 0.014 | 1.188* | 0.019 |
|  | *QLfwidth.csdh-1B.1* | *tPt-0325* | *3.3* | *1BS sat 0.50-1.00* | 0.878 | I | 0.018 | 1.000* | 0.016 |
|  | *QLfwidth.csdh-1B.2* | *psr162* | *101.0* | 1BL 0.69-0.85 | *0.683* | I, II | 0.014 | 1.063* | 0.017 |
|  | *QLfwidth.csdh-2A* | *wPt-6687* | *152.9* | 2AL 0.85-1.00 | *0.844* | III | 0.017 | 1.188* | 0.019 |
|  | ***QLfwidth.csdh-3A*** | **psr345.2** | **23.3** | **3AS 0.71-1.00** | **1.221** | **II, III** | **0.024** | **1.438**** | **0.023** |
|  | *QLfwidth.csdh-3B.1* | *wmc231* | *87.1* | 3BS 0.57-0.78 | *0.717* | - | 0.014 | 1.000* | 0.016 |
|  | ***QLfwidth.csdh-3B.2*** | ***psp3144*** | ***107.3*** | **3BL C-0.22** | ***0.829*** | **II, III** | **0.017** | **1.313*** | **0.021** |
|  | ***QLfwidth.csdh-3D.1*** | ***wPt-4569*** | ***38.9*** | **3DS 0.55-1.00** | ***-0.980*** | **I, II** | **-0.020** | **-1.125*** | **-0.018** |
|  | *QLfwidth.csdh-3D.2* | *gwm341* | *90.2* | 3DL C-0.27 | *-0.847* | I, II | -0.017 | -1.000* | -0.015 |
|  | *QLfwidth.csdh-3D.3* | *wPt-730935* | *210.5* | 3DL 0.81-1.00 | *0.879* | I | 0.018 | 1.188* | 0.019 |
|  | *QLfwidth.csdh-4A.1* | wPt-3638 | 102.3 | 4AL 0.59-0.66 | 1.043 | II | 0.021 | 1.250* | 0.020 |
|  | ***QLfwidth.csdh-4A.2*** | **gwm30.2** | **123.5** | **4AS C-0.63** | **1.362** | **I, II** | **0.027** | **1.625**** | **0.026** |
|  | *QLfwidth.csdh-4A.3* | *psp3028* | *150.9* | 4AS 0.76-1.00 | *0.580* | II | 0.012 | 1.250* | 0.020 |
|  | *QLfwidth.csdh-5B* | *gwm639.2* | *68.2* | 5BL 0.55-0.75 | *-0.880* | I, II | -0.018 | -1.125* | -0.018 |
|  | *QLfwidth.csdh-5D.1* | gwm205 | 35.2 | 5DS C-0.63 | 1.022 | II, III | 0.020 | 1.250* | 0.020 |
|  | *QLfwidth.csdh-5D.2* | gwm292 | 158.2 | 5DL 0.76-1.00 | -1.035 | II | -0.021 | -1.438** | -0.023 |
|  | *QLfwidth.csdh-5D.3* | *m77p64.10* | *234.3* | 5DL 0.76-1.00 | *-0.814* | II | -0.016 | -1.250* | -0.02 |
|  | *QLfwidth.csdh-6A.1* | *wPt-3524* | *11.6* | 6AS 0.65-1.00 | *-0.714* | III | -0.014 | -1.125* | -0.018 |
|  | ***QLfwidth.csdh-6A.2*** | **wPt-667844** | **34.3** | **6AS C-0.35** | **-1.313** | **I, II, III** | **-0.026** | **-1.813***** | **-0.029** |
|  | *QLfwidth.csdh-6A.3* | wmc256 | 74.8 | 6AL 0.55-0.90 | -1.199 | II, III | -0.024 | -1.688*** | -0.027 |
|  | ***QLfwidth.csdh-6B.1*** | **wmc397** | **73.1** | **6BL C-0.13** | **-1.359** | **I, II, III** | **-0.027** | **-1.750***** | **-0.028** |
|  | *QLfwidth.csdh-6B.2* | m43p78.13 | 92.0 | 6BL 0.40-1.00 | -1.110 | II, III | -0.022 | -1.500** | -0.024 |
|  | *QLfwidth.csdh-7B* | *wPt-4230* | *69.0* | 7BL C-0.33 | *0.758* | - | 0.015 | 1.000* | 0.016 |
|  | *QLfwidth.csdh-7D* | *wPt-7508* | *112.9* | 7DS C-0.15 | *0.801* | II | 0.016 | 1.188* | 0.019 |
| **Leaf area**  (2.274)^§^  (0.540) | *QLfarea.csdh-1A* | *m69p78.4* | *123.6* | 1AL 0.61-1.00 | *0.985* | II | 2.240 | 1.196* | 0.646 |
|  | *QLfarea.csdh-1B.1* | wPt-8682 | 27.7 | 1BS 0.84-1.06 | 1.090 | I, II | 2.479 | 1.228* | 0.663 |
|  | *QLfarea.csdh-1.B.2* | m43p78.7 | 47.4 | 1BS C-0.50 | 1.124 | I, II | 2.557 | 1.233* | 0.666 |
|  | *QLfarea.csdh-2B* | wPt-7619 | 55.8 | 2BS C-0.53 | -1.030 | I | -2.342 | -1.093* | -0.590 |
|  | ***QLfarea.csdh-3A.1*** | **m60p64.5** | **21.1** | **3AS 0.45-1.00** | **1.175** | **II, III** | **2.671** | **1.446**** | **0.781** |
|  | *QLfarea.csdh-3A.2* | *wPt-2478* | *42.6* | 3AS 0.45-1.00 | *0.895* | III | 2.034 | 1.028* | 0.555 |
|  | *QLfarea.csdh-3A.3* | *cfa2163a* | *76.6* | 3AS 0.45-1.00 | *0.880* | - | 2.001 | 1.006* | 0543 |
|  | *QLfarea.csdh-3B.1* | *wmc231* | *87.1* | 3BS 0.57-0.78 | *0.818* | - | 1.860 | 1.172* | 06.33 |
|  | *QLfarea.csdh-3B.2* | *psp3144* | *107.3* | 3BL C-0.22 | *0.877* | II, III | 1.995 | 1.326** | 0.716 |
|  | *QLfarea.csdh-3D.1* | wPt-4569 | 38.9 | 3DS 0.55-1.00 | -1.128 | I, II, III | -2.564 | -1.285* | -0.694 |
|  | *QLfarea.csdh-3D.2* | *wPt-741507* | *85.9* | 3DS 0.55-1.00 | *-0.962* | I | -2.188 | -1.041* | -0.562 |
|  | *QLfarea.csdh-4A.1* | *wPt-3638* | *102.3* | 4AL 0.59-0.66 | *0.919* | II | 2.089 | 1.093* | 0.590 |
|  | ***QLfarea.csdh-4A.2*** | **gwm30.2** | **123.5** | **4AS C-0.63** | **1.446** | **I, II, III** | **3.288** | **1.817***** | **0.981** |
|  | *QLfarea.csdh-4A.3* | *psp3028* | *150.9* | 4AS 0.76-1.00 | *0.900* | II | 2.047 | 1.274* | 0.688 |
|  | *QLfarea.csdh-4B.1* | *psp3163* | *54.9* | 4BS 0.37-0.57 | *0.998* | I | 2.269 | 1.124* | 0.607 |
|  | ***QLfarea.csdh-4B.2*** | **psp3030.2** | **77.5** | **4BL C-0.71** | **1.518** | **I, II, III** | **3.451** | **1.706***** | **0.921** |
|  | *QLfarea.csdh-4B.3* | *barc163* | *95.4* | 4BL C-0.71 | *0.920* | II | 2.092 | 1.241* | 0.670 |
|  | *QLfarea.csdh-4B.4* | *wPt-3326* | *107.1* | 4BL 0.86-1.00 | *0.952* | II | 2.164 | 1.230* | 0.664 |
|  | *QLfarea.csdh-5A* | *wPt-9094* | *60.4* | 5AL 0.57-0.78 | *0.955* | I, II | 2.171 | 1.213* | 0.655 |
|  | *QLfarea.csdh-5D.1* | *cfd7* | *135.8* | 5DL 0.60-0.74 | *-0.728* | - | -1.656 | -1.080* | -0.583 |
|  | ***QLfarea.csdh-5D.2*** | **gwm212** | **162.7** | **5DL 0.76-1.00** | **-1.295** | **I, II, III** | **-2.945** | **-1.728***** | **-0.933** |
|  | *QLfarea.csdh-6A* | *wPt-667844* | *34.3* | 6AS C-0.35 | *-0.890* | II, III | -2.024 | -1.241* | -0.670 |
|  | ***QLfarea.csdh-6B.1*** | **wmc397** | **73.1** | **6BL C-0.13** | **-1.042** | **I, III** | **-2.370** | **-1.222*** | **-0.660** |
|  | *QLfarea.csdh-6B.2* | *m43p78.13* | *92.0* | 6BL 0.40-1.00 | *-0.619* | III | -1.407 | -1.033* | -0.558 |
|  | *QLfarea.csdh-7B* | *tPt-7360* | *15.8* | 7BS 0.27-1.00 | *0.812* | I | 1.847 | 1.057* | 0.571 |
|  | *QLfarea.csdh-7D* | *wPt-7508* | *112.9* | 7DS C-0.15 | *0.935* | II | 2.126 | 1.237* | 0.668 |
| **Midrib thickness**  (0.112)^§^  (0.112) | *Qthickm.csdh-1A.1* | wPt-3904 | 30.9 | 1AS 0.86-1.00 | 1.250 | III |  | 1.250* | 0.140 |
|  | *Qthickm.csdh-1A.2* | psr1327 | 58.2 | 1AL C-0.17 | 1.116 | III |  | 1.116* | 0.125 |
|  | *Qthickm.csdh-1A.3* | psr325.1 | 68.9 | 1AL C-0.17 | 1.420 | III |  | 1.420** | 0.159 |
|  | *Qthickm.csdh-1B* | wPt-4532 | 143.8 | 1BL 0.85-1.00 | 1.054 | III |  | 1.054* | 0.118 |
|  | ***Qthickm.csdh-2D*** | **gwm102** | **75.5** | **2DS 0.33-0.47** | **1.277** | **III** |  | **1.277*** | **0.143** |
|  | *Qthickm.csdh-3A.1* | psr345.2 | 23.3 | 3AS 0.71-1.00 | 1.500 | III |  | 1.500** | 0.168 |
|  | *Qthickm.csdh-3A.2* | wPt-2478 | 42.6 | 3AS 0.45-1.00 | 1.545 | III |  | 1.545** | 0.173 |
|  | *Qthickm.csdh-3D* | dupw173 | 36.8 | 3DS 0.55-1.00 | -1.196 | III |  | -1.196* | -0.134 |
|  | ***Qthickm.csdh-4A*** | **barc78** | **32.9** | **4AL 0.80-1.00** | **1.036** | **III** |  | **1.036*** | **0.116** |
|  | *Qthickm.csdh-4B.1* | psp3163 | 54.9 | 4BS 0.37-0.57 | 1.179 | III |  | 1.179* | 0.132 |
|  | *Qthickm.csdh-4B.2* | wPt-3326 | 107.1 | 4BL 0.86-1.00 | 1.063 | III |  | 1.063* | 0.119 |
|  | *Qthickm.csdh-4D* | psp3103 | 61.9 | 4DS C-0.53 | 1.616 | III |  | 1.616** | 0.181 |
|  | *Qthickm.csdh-5A.1* | wPt-4131 | 4.6 | 5AS C-0.40 | -1.000 | III |  | -1.000* | -0.112 |
|  | *Qthickm.csdh-5A.2* | wPt-7255 | 104.8 | 5AL 0.57-0.78 | -1.143 | III |  | -1.143* | -0.128 |
|  | *Qthickm.csdh5A.3* | cfa2163b | 113.2 | 5AL 0.57-0.78 | -1.277 | III |  | -1.277* | -0.143 |
|  | *Qthickm.csdh-5D.1* | m77p64.8 | 148.3 | 5DL 0.60-0.74 | -1.170 | III |  | -1.170* | -0.131 |
|  | *Qthickm.csdh-5D.2* | gwm212 | 162.7 | 5DL 0.76-1.00 | -1.161 | III |  | -1.161* | -0.130 |
|  | *Qthickm.csdh-6D* | cfd80 | 75.8 | 6DL C-0.29 | -1.598 | III |  | -1.598** | -0.179 |
|  | *Qthickm.csdh-7B* | tPt-7360 | 15.8 | 7BS 0.27-1.00 | 1.161 | III |  | 1.161* | 0.130 |
|  | *Qthickm.csdh-7D* | barc154 | 69.7 | 7DS 0.61-1.00 | -1.241 | III |  | -1.241* | -0.139 |

^§^ Numbers in parentheses below trait names are minimum absolute additive effects for *P*<0.05 significance with LRmapqtl. The upper value refers to the

mean of minimum significant additive effects for each experiment [Method 1]. The lower value refers to the minimum significant additive effect using the 3-year mean phenotypic data for a single analysis with LRmapqtl [Method 2] (only experiment III, 2009 for midrib thickness).
