# Supplementary material for: Genetic analysis of water loss of excised leaves associated with drought tolerance in wheat
Source: PeerJ. 2018 Jul 6;6:e5063. doi: 10.7717/peerj.5063 (PMC6037134; doi:10.7717/peerj.5063)
Supplement: Table S4 [file peerj-06-5063-s005.doc]

Supplementary Table: Ratios of yield/plant between the 10 CSDH lines with the greatest leaf length or leaf area and yield/plant for the remaining 84 CSDH lines for five measures of yield/plant. Significant differences are indicated by ratios in bold italics^§^, and ratios less than one are shown in red.

| Trait | Year | Mean of all 52 trials | Mean of 19 control trials | Mean of 12 droughted trials | Site yield of  7 g/plant | Site yield of  2 g/plant |
| --- | --- | --- | --- | --- | --- | --- |
| Leaf length | 2007 | 0.978 | 0.943 | 1.026 | ***0.893***** | 0.993 |
|  | 2008 | 1.028 | 1.024 | 1.049 | 0.976 | 1.016 |
|  | 2009 | 1.054 | 1.028 | 1.077 | 0.983 | 1.040 |
|  | mean | 1.0200 | 0.9983 | ***1.0507***** | 0.9507 | 1.0163 |
| Mean leaf length | 2007-2009 | 1.020 | 0.992 | 1.033 | 0.964 | 1.009 |
| Leaf area | 2007 | 0.988 | 1.015 | 1.013 | 0.953 | 1.022 |
|  | 2008 | 1.060 | 1.060 | 1.044 | 1.004 | 1.041 |
|  | 2009 | 1.024 | 1.058 | 1.004 | 1.033 | 1.031 |
|  | mean | 1.0240 | ***1.0443******* | 1.0203 | 0.9967 | ***1.0313***** |
| Mean leaf area | 2007-2009 | 1.067 | 1.089 | 1.066 | 1.034 | ***1.065**** |

^§^ Significance of differences in yield/plant between the selected 10 CSDH lines and the remaining 84 lines for the 52 trials, 19 control and 12 droughted trials were tested using two-way ANOVA, with experiments as replications. Significance of differences in yield/plant between the selected 10 CSDH lines and the remaining 84 lines at site yields of 7 and 2 g/plant were tested for yearly mean data (row 4 for each trait) using a two-sample *t*-test with equal variances, and for three-experiment means (row 5 for each trait) using a two-sample *t*-test with unequal variances.

*, **, ***, **** Means of yield/plant for 10 CSDH lines and the remaining 84 lines significantly different at *P*<0.05, 0.01, 0.001, 0.0001, respectively,
